# Transmission of Tuberculosis through Meat and Milk1Presented at the Thirty-eighth Annual Convention of the American Veterinary Medical Association, Atlantic City, N. J., September, 1901.

**Published:** 1901-11

**Authors:** John J. Repp

**Affiliations:** Ames, Iowa, Professor of Pathology and Therapeutics, and Veterinarian to the Experimental Station, Iowa State College


### THE JOURNAL

OF

### COMPARATIVE MEDICINE AND VETERINARY ARCHIVES.

Vol. XXII. *NOVEMBER, 1901.* No. 11.

### TRANSMISSION OF TUBERCULOSIS THROUGH MEAT AND MILK.[1]

By John J. Repp, V.M.D.,

AMES, IOWA,

PROFESSOR OF PATHOLOGY AND THERAPEUTICS, AND VETERINARIAN TO THE EXPERIMENTAL STATION, IOWA STATE COLLEGE.

With the classical experiments of Villemin and Chauveau, made over thirty years ago, the belief arose among the leading students of the subject of tuberculosis that this disease of the various species of mammalian animals is the same as tuberculosis of the human species, and that it is capable of transmission among all of these species. The discovery of the germ of tuberculosis caused many more, including the discoverer, Koch, to adopt this belief, although Koch tells us he did so with reserve; and, moreover, to refer the causation of the disease in all the species above mentioned to the same tubercle bacillus. This opinion met with ready and almost universal acceptance up to the last three or four years. During this time the question of the identity of the tubercle bacillus as found in animal and in man, and the transmissibility of tuberculosis among these species, has been brought under considerable debate, not only by the laity but by scientists as well, and work has been done by investigators with a view to arriving at some definite and thoroughly defensible conclusion.

A few scientific men have recently expressed themselves in such a way as to lead one to infer that they incline to the opinion that the tubercle bacillus as found in man is radically different from that found in cattle, and that tuberculosis is not transmissible from cattle to man. None, however, have by their statements attracted

[1] Presented at the Thirty-eighth Annual Convention of the American Veterinary Medical Association, Atlantic City, N. J., September, 1901.

so much attention as did Professor Robert Koch, of Berlin, at the British Congress on tuberculosis, held recently in London, England. Professor Koch there expressed the belief that tuberculosis in man is different from tuberculosis in cattle, and cannot be transferred to cattle; and, from another consideration, that cattle tuberculosis is most likely not transferable to man to any appreciable extent. More will be said in regard to Professor Koch's view of this subject later in this article.

On account of the revival of this important consideration and the bearing it has upon sanitation, it is highly desirable that a careful statement of the facts of the case should be made and that any warrantable conclusions be adduced therefrom. This discussion will be confined to the limits indicated by the title set forth, and only the meat and the milk of the bovine species will be considered.

For the sake of clearness the subject may be treated under two general subdivisions, viz., (1) Transmission to Animals, and (2) Transmission to Man.

### I.—Transmission to Animals.

### *A. By Meat.*

The portions of the body not commonly used for food, such as the brain, large lymphatic glands, spleen, bladder, uterus, lungs, testicles, mammary gland, skin, shall be excluded. The tongue, muscles, small intermuscular lymphatic glands, digestive tube, heart, liver, and kidneys are included.

No reference will be made to experiments in which the material inoculated or fed is not such as we have chosen to consider, or in which the character of material is not stated, of which classes there are a great many experiments which, by their positive results, demonstrate that tuberculosis can be transmitted from animal to animal of the same or of a different species.

1. By Artificial Methods. Arloing[1] inoculated the muscle-juice of ten tuberculous cows into guinea-pigs, and the result showed that the muscle-juice of two of the cows was infecting, three out of ten guinea-pigs, 30 per cent., inoculated from these two cows becoming tuberculous.

Galtier[2] infected two rabbits with the juice of the flesh of a tuberculous cow.

Nocard[3] produced tuberculosis in one case out of twenty-one, 5 per cent., by injecting juice of the flesh of twenty-one cows condemned at the abattoirs on account of tuberculosis.

Woodhead[4] produced tuberculosis by injecting into the peritoneal cavity of two rabbits the raw juice from the intercostal muscles of a tuberculous cow after the tuberculous pleura had been stripped off.

Veyssiere and Humbert[5] inoculated two rabbits with 1 c.c. of flesh-juice from a tuberculous cow in very good condition, and both became tuberculous, 100 per cent.

2. By Natural Methods. Johne[6] fed thirty-five animals with the raw flesh from animals attacked with tuberculosis, and eight, or 22.5 per cent., became tuberculous.

Gerlach[7] fed forty-six subjects in the same manner, and six, or 13.1 per cent., contracted the disease.

Peuch[8] caused two young pigs to consume five pounds of raw flesh, without bone, in ten days, and in two and three months, respectively, these animals presented discrete glandular tuberculosis, 100 per cent.

Nocard[9] failed to produce tuberculosis in any of several litters of kittens which he fed with meat of cattle condemned on account of tuberculosis.

Perroncito[10] had like negative results in the case of eighteen young pigs fed from three to five months on flesh of condemned cattle.

Galtier[11] reports that a calf, aged six weeks, ate in one month four kilogrammes of raw meat taken from cows which had been seized on account of generalized tuberculosis; another calf, aged four or five months, also ate at three different times large quantities of raw tuberculous meat; two pigs, aged five to six months, ate considerable quantities of condemned meat. None of these animals developed tuberculosis. It is noteworthy that the presence of tubercle bacilli in some of this meat had been proven by inoculation of rabbits with its juice.

In speaking of the transmission of tuberculosis to animals through meat we must not lose sight of the fact that in the process of removal of the tubercular organs not ordinarily used as food, and which are for this reason not considered here, and in the cutting up of the carcass some of the food parts of the carcass may be smeared with material rich in tubercle bacilli. Such contaminated meat would, of course, be much more highly infectious. It is also to be noted that the muscles of swine and their intermuscular lymph glands are more apt to be tuberculous than is the case with similar bovine structures. An examination of the literature to which I have had access indicates that but very little experimentation has been done to determine the infectiousness of those organs and tissues of tuberculous animals which are used for food. Much experimentation has been done with tissues other than food tissues, but chiefly for the purpose of determining the general infectiousness of the disease. If these experiments with the food tissues should be extended, there is no reason to doubt that they would indicate that this is a more extensive source of infection than is at present apparent. Also, none of the experiments cited, nor any other, so far as I can learn, have been made with the liver as the object fed. German slaughter-house statistics[12] collected during 1888 and 1889 show that in all tuberculous cattle examined the liver showed the disease in 88 per cent. of cases. If a series of experiments were made by feeding this organ a much higher rate of infection would be observed. It will be noted that this is classed as a food organ.

The experiments referred to nearly all have to do with the meat of *highly* tuberculous cattle. In these cases it is quite probable that the tubercle bacillus was carried into the muscular system through the blood vascular system, it being a well-recognized fact that in generalized tuberculosis the blood contains the micro-organisms of the disease. It is quite unlikely that the food organs and tissues in mild, localized cases would contain the bacillus, unless it might be the liver, with a possible rare occurrence in some of the other organs or tissues under consideration. The food organs or tissues of these mild cases ought not to be found infectious unless contaminated from non-food organs or tissues in slaughtering or dressing. We have no evidence to show that the food parts of these mild, localized cases would be unsafe for food purposes if the non-food organs and tissues and all tuberculous food tissues were removed at the time of slaughter, provided that no contamination by smearing would take place.

It is well known that heating to a temperature of 158° F., and maintaining the temperature at this point for fifteen minutes, will devitalize the tubercle bacillus. With this in view we are led to say that thorough cooking will render inert any tubercle bacilli which may be in any way connected with a piece of meat. But the cooking must be so thorough as to subject the interior of the piece of meat to this heat for this length of time. Toussaint[13] found broiled steak infected after heating its interior to 176° F. It is presumed that he did not maintain this heat long enough Woodhead[14] found that rolls of meat weighing over four pounds which he had smeared with tuberculous matter were not sterilized by ordinary roasting and boiling.

Johne[15] in sixty-two experiments administered notoriously tuberculous flesh after it had been submitted to cooking in boiling water for ten to fifteen minutes, and 35.5 per cent. of the animals became affected.

*B. By Milk.*

This will be understood to mean milk and all of its products, as skim-milk, buttermilk, cream, butter, and cheese. The right is reserved to refer to any of these products as milk. The number of experiments and observations showing that tuberculosis may be transmitted from animal to animal of the same or different species through the medium of the milk of tuberculous cows is very great. In order to reinforce the assertion by the facts in the case a number of experiments and observations will be presented.

1. By Artificial Methods. There is no attempt made in the following presentation to separate the experiments made with milk of cows with healthy udders from those made with the milk of cows with diseased udders, but attention is invited to the fact that the milk of cows with healthy udders is, in a large number of cases, infectious for animals.

Ernst[16] reports that out of eighty-eight guinea-pigs inoculated from fifteen cows, twelve, 13.6 per cent., became tuberculous; of ninety rabbits, six, 6.6 per cent., became tuberculous.

Hirshberger[17] produced tuberculosis in fourteen out of a number of rabbits inoculated with the milk of twenty-nine tuberculous cows with sound udders.

Bang,[18] by inoculation of guinea-pigs and rabbits, found the milk of nine out of sixty-three cows with apparently healthy udders infectious. On microscopical examination, post-mortem, three of these cows showed slight udder lesions.

Peuch[19] produced tuberculosis in all four rabbits inoculated from the milk of a cow with tuberculous udder. A two-months-old pig fed five days with four and one-half quarts of milk from the same udder did not show any lesions of tuberculosis when killed fifty-six days later.

Ravenel[20] experimented as follows: Five grade cows which did not show udder infection were selected and 10 c.c. of the milk of each was at different times inoculated into the peritoneal cavity of eighty-eight guinea-pigs, of which eleven, 12.5 per cent., became tuberculous. No preparation of the milk was made, but it was taken just as it came from the cow. In another series of fifty-two guinea-pigs given the whole unprepared milk of the entire herd of five cows, no tuberculosis developed.

Russell[21] inoculated seventeen guinea-pigs with 0.5 c.c. to 4 c.c. of milk of seven different tuberculous cows, only one of which showed tuberculosis of the udder. The two, 11.8 per cent., of these seventeen guinea-pigs which were inoculated with the milk of the cow with tuberculous udder developed well-marked tuberculosis, although only 2 c.c. and 1 c.c., respectively, of milk was used.

Delepine[22] injected the sediment obtained by centrifugation of samples of country and town dairies into the subcutaneous tissues or the peritoneal cavity of sixty-five guinea-pigs, and produced tuberculosis in one of them, 0.15 per cent.

Schroeder[23] inoculated forty guinea-pigs with the sediment resulting from centrifugalizing nineteen samples of dairy and dealers' milk. Four died of intercurrent disease, and out of the thirty-six remaining, one developed tuberculosis, 0.28 per cent. Nothing is known of the cows from which the milk was obtained. In another series thirty-two guinea-pigs were inoculated intraperitoneally with centrifugalized milk of thirteen cows either known to be tuberculous or which gave a tuberculin reaction. Of these, one developed tuberculosis, 0.3 per cent. In still another series the centrifugalized or untreated milk of four cows whose udders were not diseased was injected into sixteen guinea-pigs, each receiving intraperitoneally five doses on as many different dates. None of them developed tuberculosis.

Bollinger[24] produced tuberculosis in experimental animals with the milk of a cow with non-tuberculous udder.

May[25] injected the milk of six cows, one of which had a tuberculous udder, and found the milk of the tuberculous udder infectious.

Stein[26] obtained by the same method four positive results out of fourteen injections with milk from sound udders, 28.57 per cent.

Nocard[27] inoculated the milk of fifty-four cows, and obtained infection with three which had diseased udders.

Kanthack and Sladen,[28] in examining sixteen sources of the milk-supply to the colleges in Cambridge, England, inoculated ninety guinea-pigs, with the result that twenty-three, or 25.55 per cent., died of tuberculosis. Of these twenty-three, thirteen were inoculated from the creamy layer and ten from the sediment. Of the sixteen dairies examined, nine were infectious, 56.25 per cent.

The director of the Jenner Institute of Preventive Medicine[29]

found by animal inoculation that the milk of seventeen out of one hundred samples was able to produce tuberculosis, 17 per cent.

Adami[30] reports that he and Martin, in inoculating the milk from ten tuberculous cows whose udders were free from tuberculosis, produced tuberculosis in two guinea-pigs out of twenty-nine guinea-pigs, and twenty-six rabbits inoculated intraperitoneally, 3.6 per cent. A calf fed five months with the milk of the cow that produced the disease in these two guinea-pigs did not develop the disease.

The work of Rabinowitch and Kempner[31] is very interesting as adding strong evidence to that already obtained of infectiousness of milk of tuberculous cows, even though the udder be sound. These experimenters used the milk of fifteen cows which had reacted to the tuberculin test, in ten of which the milk was found by inoculation of guinea-pigs to be infectious, 66$\frac{2}{3}$ per cent. Of the ten cows, one showed clinical evidence of udder tuberculosis, and another showed it on microscopic examination, post-mortem. Two other cows were only slightly affected, as determined by clinical examination, and still two others showed no symptoms of the disease. The inoculations were made into the peritoneal cavity with a combination of the sediment obtained by centrifuging and the fat layer. Butter was made from the milk of these ten cows, and, by inoculating guinea-pigs with it, tuberculosis was set up in three out of four guinea-pigs inoculated, 75 per cent.

Obermüller[32] reports that Brusaferro has obtained positive results with nine samples of butter; Bang from one specimen from a tuberculous cow; Roth from two out of twenty samples from diseased cows and from the market. He also reports that as a result of his own experiments he succeeded in producing tuberculosis in four out of forty-one guinea-pigs inoculated from ten different samples of butter.

Hormann and Morgenroth[33] examined ten samples of butter, using the method of intraperitoneal inoculation of guinea-pigs. The samples contained true tubercle bacilli, as proven both by lesions produced and by cultures.

2. By Natural Methods Gerlach[34] fed young animals, calves, pigs, rabbits, etc., with milk taken from tuberculous cows, and found that some of them grew thin and died at the end of a few months, showing at the autopsy an intense tubercular infection of the organs of the abdominal cavity.

Bang[35] reports autopsy on thirty-four milk-fed calves, twenty-four, 70.6 per cent., of which showed lesions of tuberculosis, evidently produced by the ingestion of milk containing tubercle bacilli. Since then this author has made numerous similar observations.[36]

Law[37] fed three calves of healthy parents on the milk of three tuberculous cows with apparently sound udders, with the result of producing tuberculosis in all three calves, 100 per cent.

Ernst[38] fed twenty-one healthy calves on milk of tuberculous cows with healthy udders, and eight, 38 per cent. of them, became tuberculous.

Ernst[39] fed forty-eight rabbits from one to three months on the milk of healthy udders, and 24.1 per cent. were infected with tuberculosis.

Freudenreich[40] examined twenty-eight samples of mixed milk, and of this number found four which proved to be virulent when inoculated into guinea-pigs.

Bang[41] says: "In Denmark the swine are almost always fed with skim-milk, buttermilk, and whey, in addition to grain, and formerly it was noticed that when these milk foods were given raw the swine almost always suffered from tuberculosis where this disease was prevalent among cows. Since attention has been directed to this danger, tuberculosis in swine has greatly diminished in my country."

Hills and Rich[42] record the observation made by one of them, that five swine born of apparently healthy parents, and fed on skim-milk from a creamery partly supplied by tuberculous cows, were found tuberculous on autopsy. Also, that many of the pigs fed on the milk of a herd of ninety-one cattle, seventy-eight of which were tuberculous, were found to be tuberculous on post-mortem examination.

Russell[43] fed two pigs, beginning at six weeks of age, from August 23 to November 10, on separator slime received from the college creamery. None of them became tuberculous.

Bang[44] says: "In Denmark milk is often given to young or to sick horses, and in those parts of the country where this custom is frequent, tuberculosis is not rare in the horse."

McFadyean[45] says: "In a considerable proportion of cases there was a distinct history of the animals having been fed with tuberculous milk. Now, when one reflects that certainly not one horse in several hundreds is at any period of its life fed on cow's milk, the frequency with which tuberculosis has been met with in horses that have been so fed becomes very striking."

The foregoing states briefly most of what has been carefully done and recorded by way of experimentation and observation to prove that tuberculosis is communicable to other animals through the medium of the milk and the food tissues and organs of tuberculous neat cattle. In addition, there might be added other observations of less definite shape, but no less true and convincing, as for example the great lessening of tuberculosis among calves of tuberculous dams which are separated at birth and fed on sterilized milk or the milk of sound cows, as has been so abundantly shown by Bang in the prosecution of his suppressive measures in Denmark. Reference might also be made to numerous observations of veterinarians pointing to the transmission of tuberculosis from tuberculous cows through their milk to calves and swine. Enough has been done to prove beyond the peradventure of a doubt that tuberculosis may be transmitted through the milk and the food structures of tuberculous animals to the animals that consume these products or are inoculated with them. Upon this all students of the subject agree. This much has been proven. But these facts do not decide the important question at issue, viz.: whether or not tuberculosis is transmissible from animal to man, nor would they if they were multiplied *ad infinitum*. They only furnish a basis from which we may reason. For this purpose they are invaluable, as they establish the promise that the meat and milk of animals do at times contain living, virulent tubercle bacilli, capable of producing disease in other animals.

(To be continued.)

